# Clinical and Genetic Features of Chinese Patients With *NIPA1*-Related Hereditary Spastic Paraplegia Type 6

**DOI:** 10.3389/fgene.2022.859688

**Published:** 2022-04-08

**Authors:** Jun Fu, Mingming Ma, Gang Li, Jiewen Zhang

**Affiliations:** ^1^ Department of Neurological Diseases, Fuwai Central China Cardiovascular Hospital, Zhengzhou, China; ^2^ Department of Neurology, Henan Provincial People’s Hospital, Zhengzhou, China; ^3^ Center of Neurological Rare Diseases of Henan Province, Zhengzhou, China

**Keywords:** *NIPA1*, hereditary spastic paraplegia, hotspot mutation, *de novo*, epilepsy, SPG6

## Abstract

**Background:** Mutations in the *NIPA1* gene cause hereditary spastic paraplegia (HSP) type 6 (SPG6), which is a rare type of HSP with a frequency of less than 1% in Europe. To date, less than 30 SPG6 families and limited *NIPA1* mutations have been reported in different ethnic regions. The clinical features are variable.

**Methods:** We screened for *NIPA1* mutations by whole exome sequencing or next generation sequencing in 35 unrelated Chinese families with HSP. The clinical manifestations were evaluated.

**Results:** Two variants of *NIPA1* were identified in three index patients (3/35, 8.6%), two of whom carried a previously reported common variant c.316G > A (p.G106R), and the third patient harbored a novel likely pathogenic variant c.126C > G (p.N42K). Both variants were *de novo* in the three index patients. The phenotype was pure HSP in two patients and complicated HSP with epilepsy in the third one.

**Conclusion:** *NIPA1*-related HSP is more common in China than it in Europe. Both pure and complicated form of HSP can be found. The variant c.316G > A is a hotspot mutation, and the novel variant c.126C > G expands the mutational spectrum. The phenomenon of *de novo* mutations in *NIPA1* emphasizes the need to consider autosomal dominant HSP-related genes in sporadic patients.

## Introduction

Hereditary spastic paraplegia (HSP) comprises a group of clinically and genetically heterogeneous neurodegenerative disorders (Erfanian et al., 2021). Clinically, HSP is classified as pure form characterized by progressive lower limb weakness and spasticity, or complicated form with additional features (Harding, 1983). Thus far, more than 80 genes for HSPs have been identified (Erfanian et al., 2021). Mutations in the non-imprinted in Prader-Willi/Angelman syndrom 1 (*NIPA1*) gene have been identified as the cause of hereditary spastic paraplegia type 6 (SPG6) with an autosomal dominant (AD) mode of inheritance (Rainier et al., 2003). SPG6 is a very rare type of HSP, accounting for less than 1% of all ADHSP cases in Europe (Klebe et al., 2007). To date, less than 30 SPG6 families have been reported in different ethnic populations (Chen et al., 2005; Bien-Willner et al., 2006; Kaneko et al., 2006; Munhoz et al., 2006; Klebe et al., 2007; Kim et al., 2019). The phenotype was often pure form; however, complicated forms have also been described with polyneuropathy (Du et al., 2011), idiopathic generalized epilepsy (Svenstrup et al., 2011), cognitive impairment (Martinez-Lage et al., 2012), ataxia (Kim et al., 2019), or amyotrophic lateral sclerosis (ALS) (Tanti et al., 2020). The mutational spectrum of *NIPA1* is quite limited with only seven mutations reported previously, and most of the SPG6 patients harbored a hotspot mutation c.316G > A (p.G106R) (Hedera, 2013).

In this study, we screened for *NIPA1* mutations by whole exome sequencing or next generation sequencing in 35 Chinese HSP families. Finally, we identified a known variant c.316G > A (p.G106R) in two unrelated patients and a novel variant c.126C > G (p.N42K) in the third patient. Both variants were *de novo* in the three index patients. Detailed manifestations were described and a general review of *NIPA1*-related HSP was performed to elucidate the clinical and genetic features of this disease.

## Materials and Methods

### Subjects

From 2018 to 2022, we performed genetic testing for 35 unrelated Chinese patients clinically diagnosed with HSP according to the Harding’s criteria (Harding, 1983) from Henan province. All index patients and some of their relatives underwent detailed clinical evaluation. The mode of inheritance was autosomal dominant in 12 families, autosomal recessive in two families, and apparently sporadic in 21 cases with no evidence of family history. Among the 35 index patients, 13 cases presented with a complicated phenotype. Three families were finally identified to be *NIPA1-*related SPG6. This study was approved by the Ethics Committee of Henan Provincial People’s Hospital. All participants gave their written informed consent.

### Genetic Analysis

Genomic DNA was extracted from peripheral blood samples from all participants following standard procedures. Whole exome sequencing was performed on some probands using Agilent SureSelect Human All Exon 50-Mb kit (Agilent, Santa Clara, CA, United States) for exome enrichment and the Illumina HiSeq2500 platform (Illumina, San Diego, CA, United States). Next generation sequencing was also conducted on the other probands using a panel targeting more than 3,000 genes related to neurological diseases, including HSP. All identified variants were validated by Sanger sequencing. The variants with minor allele frequency (MAF) of >1% in the Single Nucleotide Polymorphism Database (dbSNP), the Genome Aggregation Database (gnomAD), Exome Aggregation Consortium (ExAC), and the 1,000 Genomes Project database (1000G) were excluded. *In silico* predictions of the functional effect of variants were performed with MutationTaster (https://www.mutationtaster.org), PolyPhen-2 (https://genetics.bwh.harvard.edu/pph2) and SIFT (https://sift.jcvi.org). Co-segregation analysis was further performed by Sanger sequencing in the family members. For *de novo* variants, paternity was confirmed by analysis of highly polymorphic unlinked microsatellite makers. The novel variants were assigned in accordance with the American College of Medical Genetics and Genomics (ACMG) standards and guidelines (Richards et al., 2015).

## Results

Genetic diagnosis of HSP was established for 25 families (25/35, 71.4%). The most frequently affected gene was *SPAST* (SPG4) *(n = 9)*, followed by *SPG7* (SPG7) *(n = 4)*, *SPG11* (SPG11) *(n = 3)*, *NIPA1* (SPG6) *(n = 3, 3/35, 8.6%)*. Additional mutations were detected in *ATL1* (SPG3A), *CYP7B1* (SPG5A), *KIAA0196* (SPG8), *ALDH18A1* (SPG9B), *KIF5A* (SPG10), and *REEP1* (SPG31) in each one patient. The clinical features and mutations were briefly summarized in the Supplementary Table S1.

### *NIPA1* Mutations

**FIGURE 1:**
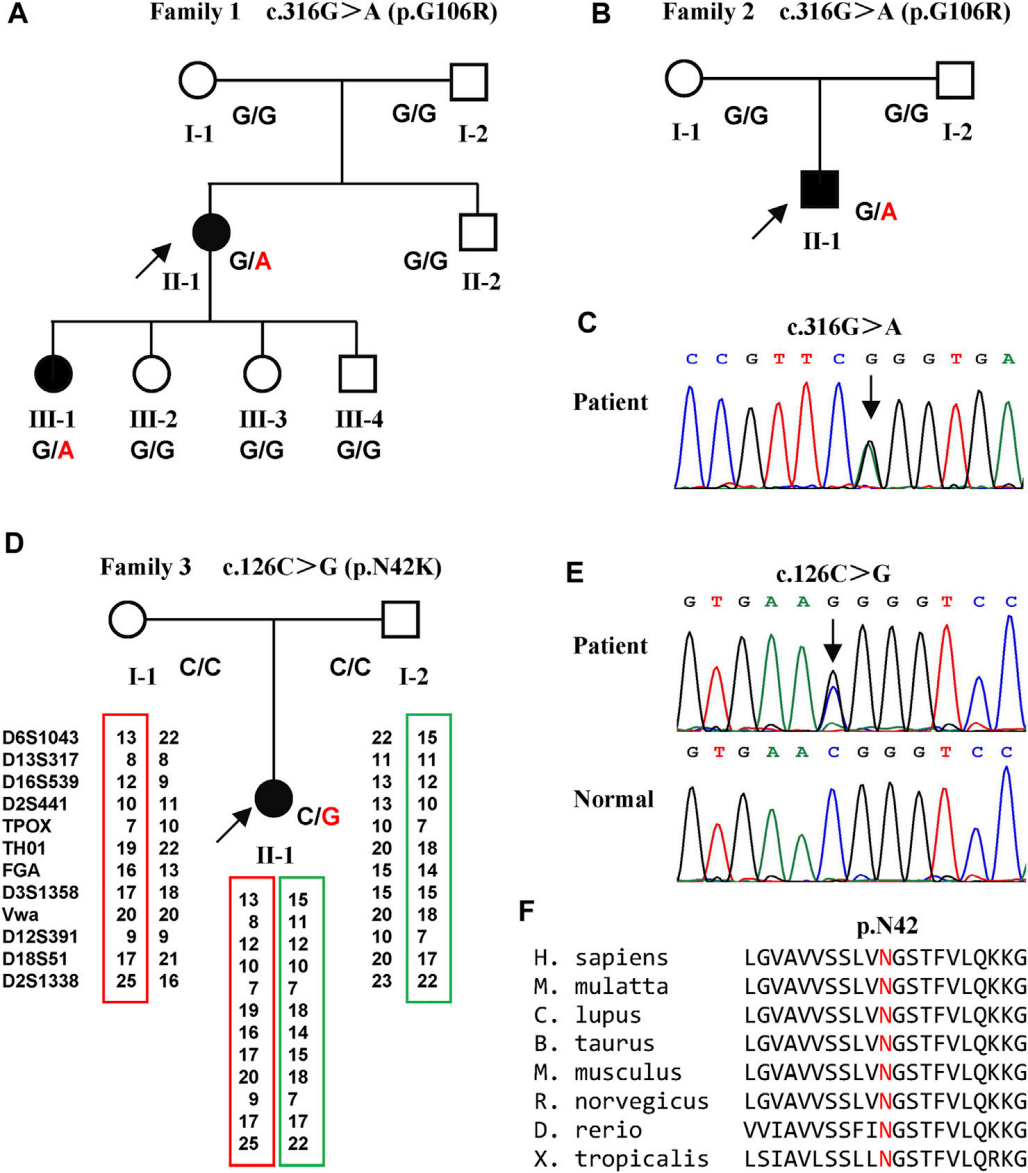
Pedigrees of the three families with SPG6 and genetic results. **(A)** Pedigree of family 1. The proband (arrow) and her elder daughter (Ⅲ-1) were heterozygous for the c.316G > A variant of *NIPA1*. **(B)** Pedigree of family 2. The proband (arrow) also carried the c.316G > A variant. **(C)** Sanger sequencing of the c.316G > A variant in the probands of family one and family 2. **(D)** Pedigree of family 3. The proband (arrow) carried the c.126C > G variant and her parents were normal. Paternity was confirmed by 12 highly polymorphic unlinked microsatellite makers. **(E)** Sanger sequencing of the c.126C > G variant in the proband of family three and her patients. **(F)** The asparagine at amino acid 42 was conserved in different species.

Two variants of *NIPA1* (NM_144599) were identified in three families (Figure 1). A previously reported heterozygous variant, c.316G > A (p.G106R) (Figure 1C) (Chen et al., 2015), was detected in two index patients (family 1 Ⅱ-1 and family 2 Ⅱ-1) (Figures 1A,B). This variant was only found in one daughter (family 1 Ⅲ-1) of the first index patient. Both parents of the two index patients did not harbor this variant, indicating that it was a *de novo* variant.

A previously unreported variant, c.126C > G (p.N42K) (Figure 1E) in exon one of *NIPA1*, was found in the third index patient (family 3 Ⅱ-1) (Figure 1D). This variant was neither found in ExAC nor 1000G, and predicted to be damaging by *in silico* analysis. The amino acid asparagine at position of 42 was conserved in different species (Figure 1F). Both parents of the third index did not harbor this variant, and true parenthood was confirmed by 12 highly informative unlinked microsatellite markers (Figure 1D). Thus, the variant c.126C > G was also *de novo*. According to the standards of ACMG, the novel variant c.126C > G was classified as likely pathogenic (evidence PS2+PM2+PP3).

### Clinical Manifestations of Patients With *NIPA1* Mutations

The index patient of family 1 (Ⅱ-1) presented with gradually progressive lower limb weakness and stiffness since the age of 23 years (Table 1). She deteriorated and was assisted by a walker in the past 2 years. A history of generalized epilepsy was reported since 10 years old. She was treated with oral valproic acid irregularly, and no seizures occurred in the past 5 years. Neurological examination at the age of 35 years revealed marked spasticity, moderate weakness, and hyperactive deep tendon reflexes that were more prominent in the lower extremities. Bilateral ankle clonus, extensor plantar responses and pes cavus were also observed. There was no impairment of cognition, sensation, sphincter or cerebellar function. Brain and spine MRI were normal. Her elder daughter (family 1 Ⅲ-1), now 15 years old, had no symptoms of spasticity, but revealed hyperactive deep tendon reflexes in the lower limbs and extensor plantar responses (Table 1). Examination of both parents was normal.

**TABLE 1:** Clinical features of affected family members carrying *NIPA1* mutations.

	Family 1		Family 2	Family 3
	Ⅱ-1	Ⅲ-1	Ⅱ-1	Ⅱ-1
Mutation	c.316G > A	c.316G > A	c.316G > A	c.126C > G
Age at examination (years)	35	15	28	17
Age at onset (years)	12	15	23	5
Epilepsy	+	−	−	−
Cognition impairment	−	−	−	−
Neuropathy	−	−	−	−
Impaired vibration sense	−	−	−	+
Bladder dysfuction	−	−	−	−
Upper limbs		−	−	−
Tremor	−	−	−	−
Spasticity	−	−	−	−
Weakness	−	−	−	−
Hoffmann’s sign	+	−	+	+
Hyperreflexia	+	−	++	++
Lower limbs				
Pes cavus	+	−	−	−
Spasticity	+	−	+	+
Weakness	+	−	−	−
Hyperreflexia	++	++	++	++
Clonus	+	−	+	+
Extensor plantar	+	+	+	+

+present; −absent; hyperreflexia: + brisk, ++ very brisk.

The index patient of family 2 (Ⅱ-1) was a 28 year-old man with the complaint of gradually progressive leg stiffness and shaking for 5 years (Table 1). He had no epilepsy or cognition impairment. Neurological examination revealed hyperactive deep tendon reflexes in both upper and lower limbs, bilateral ankle clonus, and extensor plantar responses. Both of his parents were normal on examination.

The index patient of family 3 (Ⅱ-1) was referred with the early onset gait disturbance since the age of 5 years (Table 1). She did not have any other medical problems and her parents were normal. Upon examination at age 17, a moderate spasticity especially in the lower limbs was found associated with mild diminished vibration sensation distally. MRI studies showed thoracic spinal cord atrophy. Electromyography and nerve conduction velocity studies were unremarkable.

## Discussion

In this study, we detected three patients with *NIPA1* mutations amongst 35 Chinese HSP families. Thus, the mutation frequency was 8.6%. *NIPA1* mutation was reported to be a rare cause of HSP (Klebe et al., 2007). Though identified in different ethnic populations (Chen et al., 2005; Kaneko et al., 2006; Bien-Willner et al., 2006; Munhoz et al., 2006; Klebe et al., 2007; Kim et al., 2019), less than 30 families with *NIPA1*-related SPG6 have been reported since the year of 2003 (Rainier et al., 2003) (Table 2). In the previous genetic screening studies of HSP, there was no *NIPA1* mutation identified in German (Beetz et al., 2008), Italian (D'Amore et al., 2018), Korean (Yang et al., 2021) or Japanese (Ishiura et al., 2014) patients, and only one case carrying *NIPA1* mutation found in France (Klebe et al., 2007), Hungarian (Balicza et al., 2016) and Danish (Svenstrup et al., 2011) patients, respectively (Table 3). However, previous studies in Chinese patients revealed a high *NIPA1* mutation frequency of 3.6% (Lu et al., 2018), and it was the third most common cause of ADHSP (Dong et al., 2018). The higher mutation rate of *NIPA1* in our study may be due to small sample size. Together with our study, *NIPA1*-related SPG6 was more common in China than it in Europe or other Asian countries.

**TABLE 2:** Clinical features and *NIPA1* mutations of SPG6 families reported in the literature and in the present study.

Mutation	Inheritance	Age at Onset (years)	Phenotype	Family Origin	Citation
c.126C > G (p.N42K)	*de novo*	5	Pure	Chinese	**this study**
c.134C > G (p.T45R)	AD	12–35	Pure	Irish	Rainier et al. (2003)
	AD	late teenage	Pure	Iraqi	Rainier et al. (2003)
c.249C > G (p.N83K)	*de novo*	early onset	Complicated (epilepsy)	Italian	Fabbro et al. (2021)
c.298G > A (p.A100T)	AD	10–49	Pure	Japanese	Kaneko et al. (2006)
c.316G > C (p.G106R)	AD	13–35	Pure	Chinese	Chen et al. (2005)
	AD	8–37	Pure/Complicated (memory deficit)	French	Klebe et al. (2007)
	AD	12–20	Pure/Complicated (polyneuropathy)	Chinese	Liu et al. (2008)
	AD	15–20	Complicated (polyneuropathy, pes cavus)	Chinese	Du et al. (2011)
c.316G > A (p.G106R)	AD	17–40	Pure	Chinese	Chen et al. (2005)
	AD	9–23	Pure/Complicated (epilepsy, cognitive impairment, tremor)	British	Reed et al. (2005)
	AD	6–10	Pure	American	Bien-Willner et al. (2006)
	AD	20–27	Pure	Brazilian	Munhoz et al. (2006)
	AD	10	Complicated (epilepsy, tremor, dysarthria, facial dystonia)	Danish	Svenstrup et al. (2011)
	AD	13	Complicated (ALS, cognitive impairment)	American	Martinez-Lage et al. (2012)
	*de novo*	5	Pure	American	Hedera, (2013)
	*de novo*	17	Pure	American	Hedera, (2013)
	*de novo*	10	Pure/Complicated (epilepsy)	American	Arkadir et al. (2014)
	AD	20	Pure	Chinese	Lu et al. (2018)
	*de novo*	1	Complicated (epilepsy, tremor, dysmetria, polyneuropathy)	Chinese	Lu et al. (2018)
	*de novo*		Pure	Chinese	Zhao et al. (2019)
	*de novo*	16	Complicated (ataxia)	Korean	Kim et al. (2019)
	AD	30	Complicated (ALS, epilepsy)	British	Tanti et al. (2020)
	*de novo*	10	Complicated (epilepsy)	Italian	Spagnoli et al. (2021)
	*de novo*	12	Complicated (epilepsy, pes cavus)	Chinese	**this study**
	*de novo*	23	Pure	Chinese	**this study**
c.731A > G (p.Q244R)	AD	1.5	Pure	Hungarian	Balicza et al. (2016)
c.748A > C (p.K250Q)	AD		Pure	Chinese	Zhao et al. (2019)

AD, autosomal dominant; ALS, amyotrophic lateral sclerosis.

**TABLE 3:** *NIPA1* mutation rate in different regions.

Region	Result	References
China	3.6% (2/55) HSP	Lu et al. (2018)
	8.6% (3/35) HSP	this study
Japan	0/129 HSP	Ishiura et al. (2014)
Korea	0/104 HSP	Yang et al. (2021)
Italy	0/239 HSP	D'Amore et al. (2018)
Gemany	0/101 HSP	Beetz et al. (2008)
Europe (France)	0.9% (1/110) ADHSP	Klebe et al. (2007)
Hungary	1.7% (1/58) HSP	Balicza et al. (2016)
Denmark	1.9% (1/52) HSP (30 ADHSP and 22 sporadic cases)	Svenstrup et al. (2011)

ADHSP, autosomal dominant hereditary spastic paraplegia.

The phenotype of our patients with SPG6 was similar to the other reports. The age of disease onset was usually in the second and third decades, although variability could also be observed (Table 2). The index patient of family three in our study had an early onset age of 5 years, and it could be as early as 1 year (Lu et al., 2018). The disease often progressed slowly, while some patients deteriorated and required walking aids in their twenties or thirties as the first index patient in our study (Bien-Willner et al., 2006; Svenstrup et al., 2011; Hedera, 2013). SPG6 is known as a generally pure form of HSP; however, more cases with a complicated phenotype have also been reported. The co-morbidities included idiopathic generalized epilepsy (Svenstrup et al., 2011), polyneuropathy (Liu et al., 2008; Du et al., 2011), cognitive impairment (Martinez-Lage et al., 2012), ataxia (Kim et al., 2019), postural tremor (Svenstrup et al., 2011; Lu et al., 2018) and amyotrophic lateral sclerosis (ALS) (Tanti et al., 2020). Until now, epilepsy has been described in eight families with SPG6 (Reed et al., 2005; Svenstrup et al., 2011; Arkadir et al., 2014; Lu et al., 2018; Tanti et al., 2020; Fabbro et al., 2021; Spagnoli et al., 2021), including the first index patient in our study, who presented with a complicated form of HSP. Why *NIPA1* mutation might cause epilepsy is unclear. The other patients in the present study showed a pure form of HSP.

**FIGURE 2:**
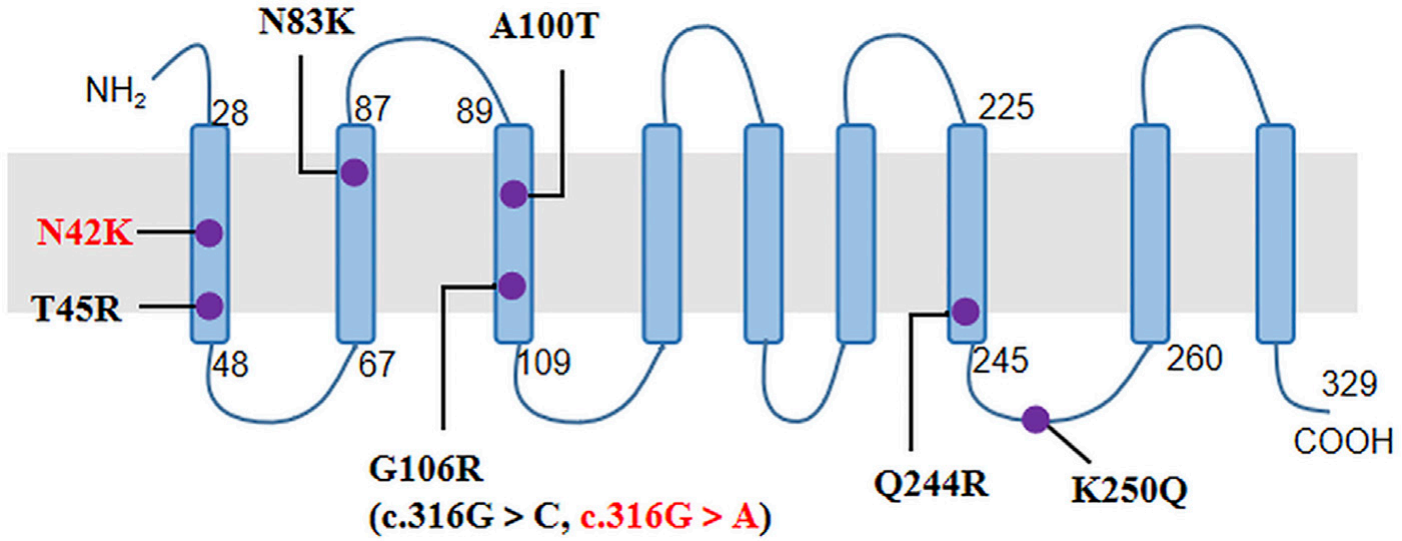
The nine transmembrane domains of NIPA1 protein and localization of *NIPA1* mutations. The two mutations identified in our patients were indicated by red colour.

The *NIPA1* gene has five coding exons located at 15q11.2, and encodes a nine transmembrane protein as an intracellular magnesium transporter (Goytain et al., 2007). To date, only seven missense variants of *NIPA1* have been previously reported (Table 2; Figure 2). In our study, we identified the most common variant c.316G > A (p.G106R) in two unrelated patients. It has been discovered in more than 10 HSP families from China (Chen et al., 2005; Lu et al., 2018; Zhao et al., 2019), Britain (Reed et al., 2005), Brazil (Munhoz et al., 2006), Danmark (Svenstrup et al., 2011), Italy (Spagnoli et al., 2021), Korea (Kim et al., 2019) and America (Hedera 2013). Thus, it was considered to be a hotspot mutation of *NIPA1* with the mechanism of DNA methylation in the coding regions (Beetz et al., 2008). Patients carrying the variant c.316G > A could present with pure or complicated HSP (Tanti et al., 2020). We further identified a novel variant c.126C > G (p.N42K) in the third index patient. It occurred in the first transmembrane domain and was near to the reported pathogenic variant c.134C > G (p.T45R) (Rainier et al., 2003). By analysis of *in silico* predictions and family segregation, c.126C > G was classified as likely pathogenic; therefore, it expanded the mutational spectrum of *NIPA1*. The patient carrying this novel variant presented with a pure HSP. The disease mechanism of *NIPA1*-related SPG6 is likely to be toxic gain of function (Zhao et al., 2008).

Interestingly, all of the three index patients in our study harbored a *de novo* mutation, which was not common in HSP (Lu et al., 2018). Most of the previously reported SPG6 patients were familial cases with autosomal dominant inheritance (Rainier et al., 2003). The *de novo* mutations of *NIPA1* were only documented in several cases since the first report by Hedera (Hedera et a., 2013). In such sense, the ADHSP-related genes need to be considered in the screening of HSP patients without family history.

Recently, a meta-analysis provided evidence for the association of *NIPA1* repeat expansions with ALS, which showed an overall increased risk of ALS in those with expanded (>8) GCG repeat length (Tazelaar et al., 2019). In addition to *NIPA1*, repeat expansions in *C9orf72* and *ATXN2* have also been reported in ALS (Elden et al., 2010; DeJesus-Hernandez et al., 2011). However, *NIPA1* repeat length was not confirmed to be a modifier of the *C9orf72* ALS disease risk (Corrado et al., 2019).

In summary, we reported three SPG6 families, which indicated that *NIPA1* mutations were more common in China. The phenotype of SPG6 included both pure and complicated HSP. The variant c.316G > A of *NIPA1* was a hotspot mutation, and the novel variant c.126C > G expanded the mutational spectrum. The phenomenon of *de novo* mutations in *NIPA1* emphasized the need to consider ADHSP-related genes in sporadic patients.

## Data Availability

The datasets for this article are not publicly available due to concerns regarding participant/patient anonymity. Requests to access the datasets should be directed to the corresponding author.
