# Supplementary material for: Clinical and Genetic Features of Chinese Patients With NIPA1-Related Hereditary Spastic Paraplegia Type 6
Source: Front Genet. 2022 Apr 8;13:859688. doi: 10.3389/fgene.2022.859688 (PMC9024055; doi:10.3389/fgene.2022.859688)
Supplement: Supplementary file 1 [file Table1.DOCX]

Supplementary Material

# Supplementary Table 1 The clinical and genetic features of 25 index patients with HSP

| Patient | Sex/Age (years) | AAO (years) | Inheritance | Phenotype | Gene | Variant | Reported | Genotype |
| --- | --- | --- | --- | --- | --- | --- | --- | --- |
| 1 | F/6 | 3 | AD | Pure | *ATL1* | c.715C＞T (p.R239C) | Yes | SPG3A |
| 2 | M/38 | 3 | AD | Pure | *SPAST* | Exon 5-7 del | Yes | SPG4 |
| 3 | F/33 | 31 | AD | Pure | *SPAST* | Exon 1-3 del | Yes | SPG4 |
| 4 | M/49 | 45 | AD | Pure | *SPAST* | c.1196C＞G (p.S399W) | Novel | SPG4 |
| 5 | F/30 | 10 | Sporadic | Complicated (cerebellar atrophy) | *SPAST* | c.307_312dupTCGGCC (p.S103_A104dup) | Yes | SPG4 |
| 6 | M29 | 26 | Sporadic | Pure | *SPAST* | c.508_509delCA(p.Q170Vfs*2) | Novel | SPG4 |
| 7 | M/55 | 20 | AD | Pure | *SPAST* | c.1813dupA(p.I605Nfs*26) | Novel | SPG4 |
| 8 | M/46 | 41 | AD | Pure | *SPAST* | c.1774delA (p.I592*) | Yes | SPG4 |
| 9 | F/36 | 32 | AD | Pure | *SPAST* | c.1328_1329delinsCCTAGAG (p.V443Afs*2) | Novel | SPG4 |
| 10 | M/57 | 47 | AD | Pure | *SPAST* | c.1821G＞C (p.W607C) | Yes | SPG4 |
| 11 | M/13 | 7 | Sporadic | Complicated (white matter lesion) | *CYP7B1* | c.1162C＞T (p.R388*) /c.1171G＞T (p.D391Y) | Yes/Novel | SPG5A |
| 12 | F/17 | 5 | Sporadic | Pure | *NIPA1* | c.126C＞G (p.N42K) | Novel | SPG6 |
| 13 | M/28 | 23 | Sporadic | Pure | *NIPA1* | c.316G＞A (p.G106R) | Yes | SPG6 |
| 14 | F/35 | 12 | Sporadic | Complicated (epilepsy) | *NIPA1* | c.316G＞A (p.G106R) | Yes | SPG6 |
| 15 | F/36 | 27 | AR | Complicated (ataxia) | *SPG7* | c.2014G＞A (p.G672R) /c.2062_2063delinsTA (p.R688*) | Yes/Yes | SPG7 |
| 16 | F/46 | 41 | AR | Complicated (cerebellar atrophy) | *SPG7* | c.1150_1150+1insCTAC (p.G384Afs*13)(Hom) | Yes | SPG7 |
| 17 | M/45 | 41 | Sporadic | Complicated (cerebellar atrophy) | *SPG7* | c.1454G＞A (p.R485K) / c.1937-2A＞C | Novel/Novel | SPG7 |
| 18 | M/51 | 45 | Sporadic | Complicated (ataxia) | *SPG7* | c.2176C＞T (p.Q726*)(Hom) | Novel | SPG7 |
| 19 | M/12 | 5 | AD | Pure | *KIAA0196* | c.1128delG (p.M376Cfs*24) | Novel | SPG8 |
| 20 | F/31 | 23 | Sporadic | Complicated (neuropathy) | *ALDH18A1* | exon3 c.250C＞T (p.R84*) /c.1994G＞A (p.R665Q) | Novel/Yes | SPG9B |
| 21 | M/18 | 13 | Sporadic | Complicated (neuropathy) | *KIF5A* | c.910C＞T (p.R204W) | Yes | SPG10 |
| 22 | M/11 | 10 | Sporadic | Complicated (thin corpus callosum) | *SPG11* | c.733_734delAT (p.M245Vfs*2) /  c.3987_3988insAA（p.Q1330Nfs*4） | Yes/Novel | SPG11 |
| 23 | M/17 | 12 | Sporadic | Complicated (neuropathy) | *SPG11* | c.1825C＞T (p.Q609*) /c.2068A＞G (p.I870V) | Novel/Yes | SPG11 |
| 24 | F/32 | 30 | Sporadic | Pure | *SPG11* | c.733_734delAT (p.M245Vfs*2) /  c.3483_3484dupGT（p.F1162Cfs*10） | Yes/Novel | SPG11 |
| 25 | M/4 | 3 | AD | Pure | *REEP1* | Exon 3 del | Yes | SPG31 |

M, male; F, female; AAO, age at onset; AD, autosomal dominant; AR, autosomal recessive.
